# First Reported Case of Breast Sporotrichosis in Bahrain

**DOI:** 10.7759/cureus.74128

**Published:** 2024-11-20

**Authors:** Suhair Al Saad, Hamdi Al Shenawi, Herminia Tyminski, Nisha Chandran, Eman Farid, Noor Al Shenawi

**Affiliations:** 1 Breast Surgery, Dr. Suhair Al Saad Medical Centre, Manama, BHR; 2 Surgery, Arabian Gulf University, Manama, BHR; 3 Radiology, Al Khaleej Polyclinics, Manama, BHR; 4 Pathology and Laboratory Medicine, Salmaniya Medical Complex, Manama, BHR; 5 Pathology and Immunology, Arabian Gulf University, Manama, BHR

**Keywords:** biopsy, covid-19, culture, granulomatous, imaging, mastitis, sporothrix schenckii fungus, sporotrichosis

## Abstract

Granulomatous mastitis is a chronic inflammation of the breast, mostly of unknown etiology. The treatment would be definitive if the causative organism were isolated. It is characterized histologically by granulomas, formed mostly by polymorph nuclear neutrophils and central necrosis. Herein, we report a 46-year-old woman who presented with a non-responding to treatment right breast abscess of six months duration. The abscess was associated with multiple fistula formation. Radiological investigations were inconclusive. She needed incision and drainage many times. The diagnosis of granulomatous mastitis secondary to sporotrichosis can only be made after breast tissue biopsy and culture. She was started on itraconazole. She responded slowly and later needed resection of the remaining mass.

## Introduction

Mastitis is defined as an inflammation of the breast tissue. Its prevalence worldwide is between 1-10%. In a Cochrane review, the prevalence reached up to 33%. Mastitis primarily affects lactating females of reproductive age [1].

Sporotrichosis is a subacute or chronic mycosis of the subcutaneous layer. It is present mainly in tropical and subtropical zones and reported mostly in Latin America and North China [2,3]. There are no reported cases in Bahrain or the other Arabian Gulf countries [4].

The mechanism of infection with Sporothrix schenckii fungus is usually by traumatic inoculation through the skin after contact with contaminated soil, plants, organic matter, or animals (zoonotic transmission via scratches or bites of infected cats, dogs, or wild animals) [2,3]. It primarily affects the upper limbs, followed by the face, lower limbs, and chest, respectively [2,3]. The diagnosis is made by isolating and identifying the Sporothrix species in culture media. The other diagnostic methods are histopathology, serology, and molecular studies (polymerase chain reaction (PCR)) [2,3]. 

## Case presentation

A 46-year-old healthy Bahraini housewife, married with four children, presented with a right breast mass. The right breast mass was draining pus over the last six months. She has no significant past medical or surgical history and no family history of breast cancer. She breastfed all her children. Her last delivery was 10 years ago. She has no history of trauma, travel, or animal contact. She used to look after her garden, planting and watering.

**Figure 1:**
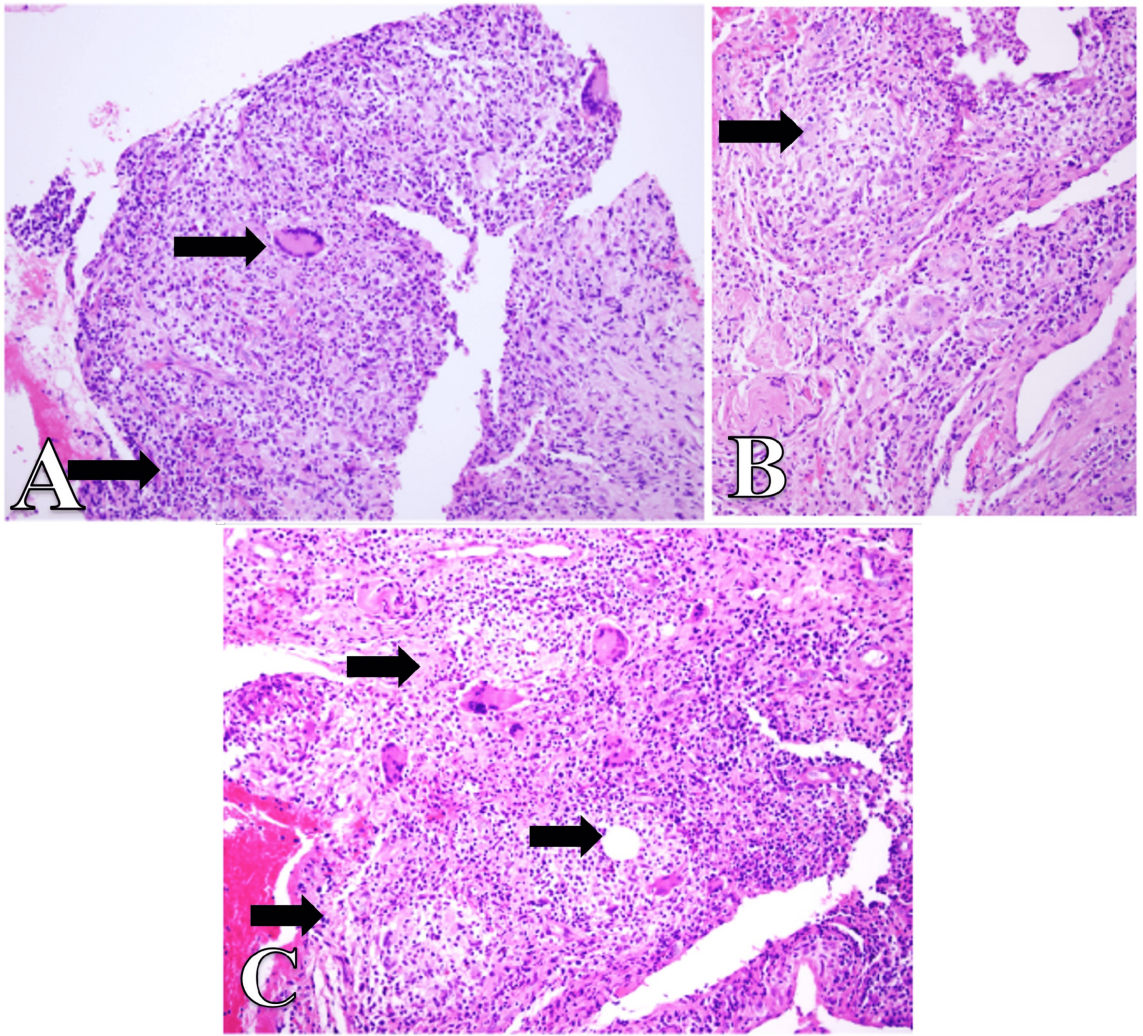
Histopathology of the Incisional Biopsy (A) Sections from breast tissue shows granulomatous inflammation. There are many epithelioid granulomatous with giant cells, H&E 10X (B) Granuloma with many neutrophils at the center, H&E 20X (C) Microneutrophilic abscesses are noted within granuloma centered around vacuolated empty spaces, H&E 40X

During the COVID-19 pandemic, she took the first dose of the COVID-19 vaccine in March 2021, and the second on 14 April 2021. She claims that her symptoms initially started after a severe COVID-19 infection in October 2021. She had a painful right breast mass that rapidly increased in size. The mass was draining pus through multiple sinuses. She underwent incision and drainage in another hospital. The histopathology of the breast tissue biopsy at that time was granulomatous mastitis with unspecific cause (Figure 1).

**Figure 2:**
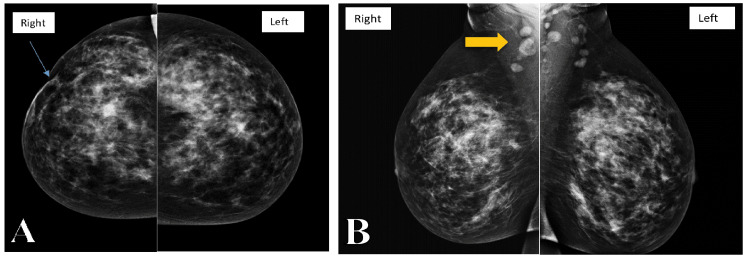
Mammogram images of craniocaudal (CC) and mediolateral oblique (MLO) views show heterogeneous breast parenchyma which limits the sensitivity of the exam. (A) CC images are significant only for mild thickening and retraction of the skin in the lateral right breast (arrow). (B) MLO images are unremarkable except for bilateral axillary lymphadenopathy more prominent in the right axilla (arrow).

**Figure 3:**
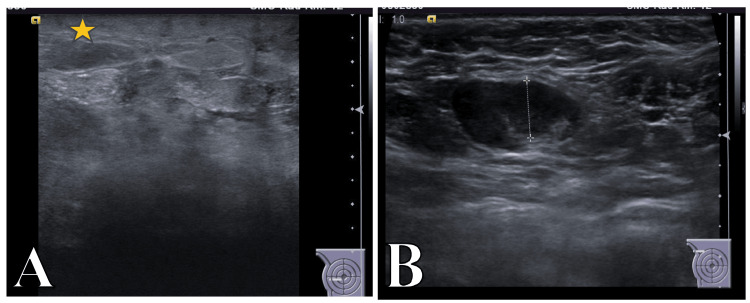
Ultrasound images (A) Ultrasound image of the right breast shows skin thickening (star) with significant subcutaneous edema leading to poor acoustic window and obscuring the underlying breast tissues. (B) Ultrasound image of the right axilla shows an enlarged lymph node with significant cortical thickening.

Again, on the first of January 2022, she received the booster dose of COVID-19; 10 days later, she got a severe COVID-19 infection, which flourished her symptoms, and the right breast mass recurred and became painful and bigger. The mammogram and breast ultrasound were inconclusive (Figures 2, 3).

**Figure 4:**
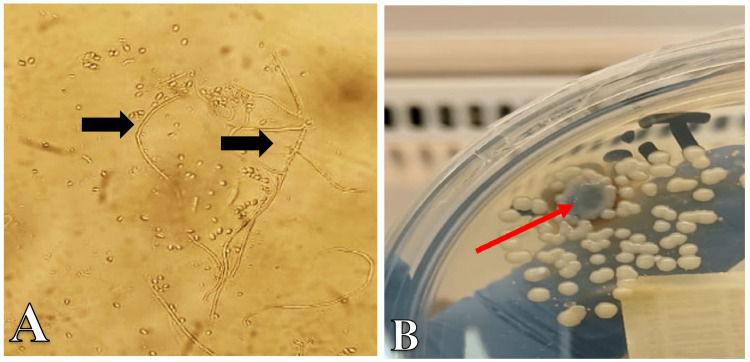
Microbiological Findings of fungal culture of tissue taken from the incisional biopsy (A) Microscopy showed Sporothrix schenckii, microscopic morphology of the thin septate hyphae with the special branching conidiophores and conidia arrangements single and in groups (shown by black arrows). (B) Cultured colonies of Sporothrix schenckii on Sabouraud dextrose agar at 37 degrees, showing yeast-like smooth white colonies, with one melanized colony with a wrinkled folded surface (shown by red arrow).

Most of the cultures were negative for bacteria, but she was still treated with empirical antibiotic courses with no significant response. On the 3rd of March 2022, she visited our surgical clinic with a huge right breast inflammatory mass occupying the central and lateral aspects (size 15x10 cm). She needed an incision and drainage. It was repeated multiple times. These abscesses were recurrent and non-communicating. All investigations for Mycobacterium tuberculosis were negative (PCR, culture, and purified protein derivative (PPD) test). A breast tissue culture in April 2022 showed delayed growth of a dimorphic fungus, Sporothrix schenckii, supporting the diagnosis of sporotrichosis (Figure 4).

**Figure 5:**
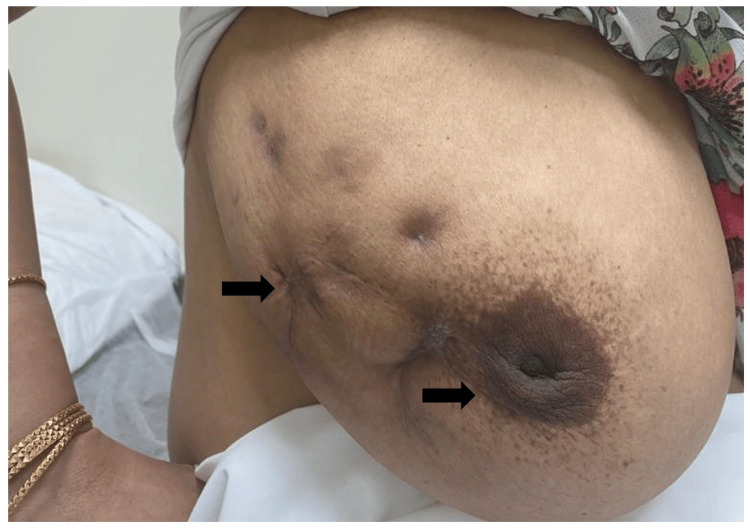
Right breast with scarred central and lateral aspect, retracted nipple and lateral displacement (November 2022)

She was started on itraconazole. The response to itraconazole was slow and it took around 10 months to become 5 cm in size, sinuses healed and the pus stopped but her nipple retracted (Figure 5).

In March 2023, a core biopsy was repeated to decide about the discontinuation of itraconazole treatment. The histopathology result was non-specific mild inflammation. We decided to go for an excision of the remaining mass.

**Figure 6:**
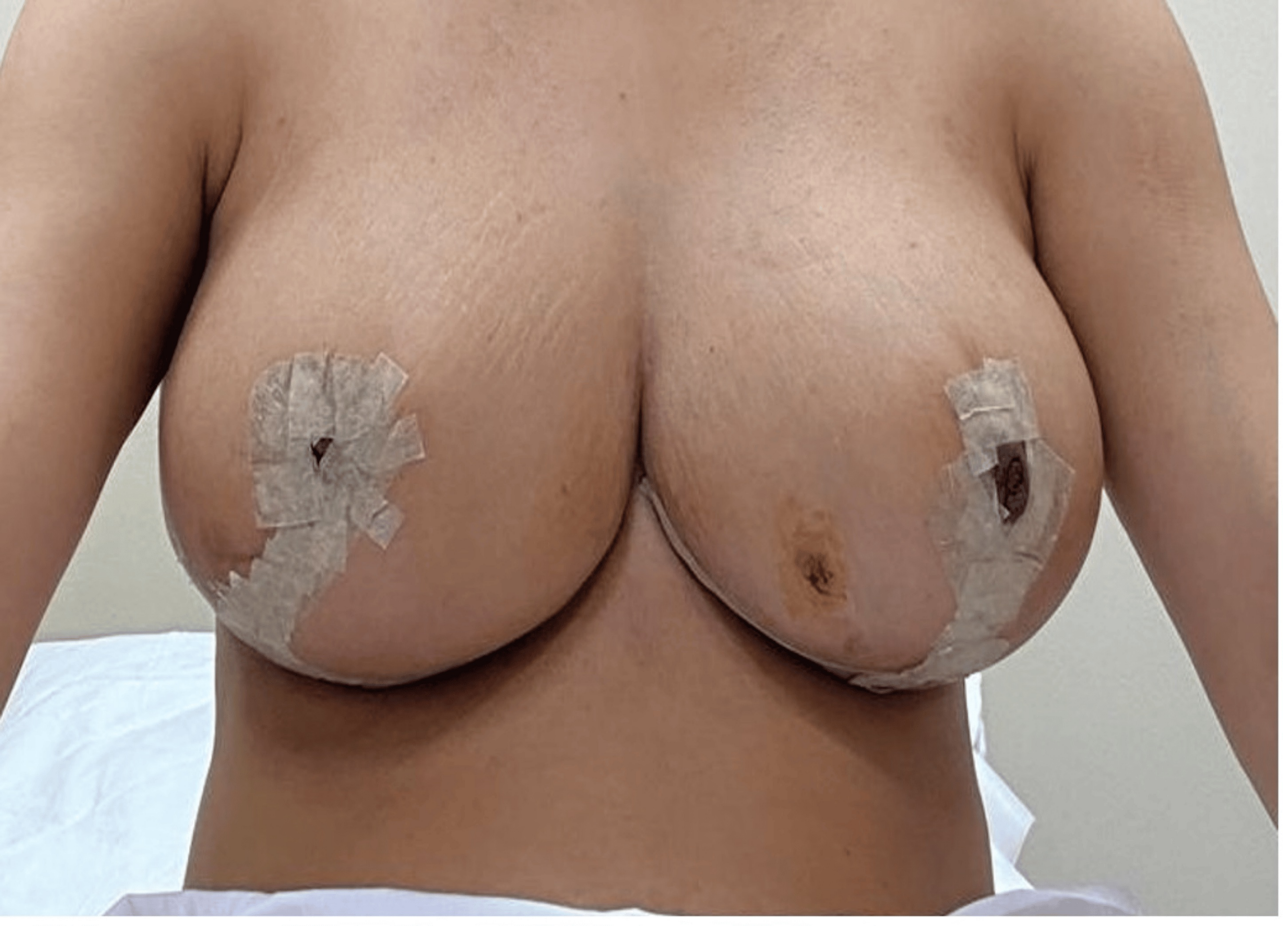
11 days postoperative

The patient chose to have excision of the right breast mass and bilateral breast reduction at the same time. The operation was uneventful, and the histopathology report indicated mild chronic inflammation (Figure 6). Follow-up one year later showed no recurrence of the infection, and the patient was satisfied with the result.

## Discussion

The etiology of mastitis is classified into two categories, septic (secondary to infection) or aseptic (idiopathic). The infection is predominantly secondary to a microorganism, like bacteria originating from the skin. Mastitis is more common in lactating ladies. It can also start from nipple lesions or after trauma. Methicillin-resistant Staphylococcus aureus (MRSA) is the most common bacteria, followed by coagulase-negative staphylococci and gram-negative bacilli. Bartonella henselae, mycobacteria, atypical mycobacteria, Actinomyces spp., Brucella spp., fungi (Candida spp. and Cryptococcus spp.) and parasites are less frequent. Polymicrobial etiology with anaerobes like Clostridium spp., Propionibacterium spp., Labacteroides spp., Veillonell spp., and Fusobacterium spp. occurs in 15-40% of cases [1,3]. Corynebacterium has also been involved in mastitis; these microorganisms can lead to breast abscesses in 0.4-11% and ductal fistulas in 1-2% [3].

The non-infection etiology is unrelated to an infective pathogen [2,3]. It could be related to autoimmune disease, sarcoidosis, or more recently reported COVID-19 infection or vaccination [5]. Granulomatous mastitis is considered within this large group of mastitis [3]. It is a chronic inflammatory disease of the breast characterized by the formation of granulomatous changes. It is clinically and radiologically challenging to differentiate from inflammatory breast cancer [6-8]. Core biopsy is an important diagnostic tool to exclude inflammatory breast cancer [8,9].

Granulomatous mastitis needs long-term medical treatment, and surgery is not the first option [8]. Treatment with antibiotics is not always helpful except if the culture is positive. Most of the time, the causative organism is difficult to discover early, like in our case, or may not be discovered at all.

A Turkish study by Isil Basara Akin reported a case of mastitis worsened after the COVID-19 vaccination. The inflammation formed an abscess and became resistant to antibiotics [10].

Another study from Mexico by Daniel D. Gallardo et al. reported the same sequela post-COVID-19 infection for a non-lactating lady who had bilateral breast implants in August 2020. One year later, she had a severe attack of COVID-19. This was followed a few days later with bilateral mastitis. They assumed that the etiology is a cross-reaction between the COVID-19 viral peak protein and antigens on the surface of the microtextured implants or related to excess production and secretion of brain-derived neurotrophic factor (BDNF) and nerve growth factor (NGF). The patient recovered well on corticosteroids [11]. In our case, the mastitis worsened after the COVID-19 infection.

Another study from Turkey reported some obstacles in using steroids as the first option in the management of idiopathic granulomatous mastitis during the COVID-19 pandemic. Still, they did not recommend withholding of steroids [12].

Sporotrichosis is a cutaneous fungal infection. The causative organism is the saprophytic mold Sporothrix schenckii [2,13,14]. Benjamin Schenck discovered sporotrichosis for the first time in 1898. Benjamin was a medical student at the Johns Hopkins Hospital in Baltimore. Hektoen and Perkins also described the fungus Sporothrix schenckii isolated from a skin lesion in 1900. In Brazil, it was first reported in 1907 by Lutz and Splendore, who also found that its symptoms are mainly cutaneous nodules, ulcers, and sometimes abscesses [15]. The first case reported in Panama was caused by cat scratches on the upper limb of a 34-year-old man reported in 2018 [16]. Diagnosis is by culture [2,17,18]. No reports exist in Bahrain, Kuwait, or the rest of the Gulf countries [4].

The standard method for diagnosis of sporotrichosis is isolating and identifying the Sporothrix species from clinical samples. Other diagnostic methods include histopathology, serology, and molecular studies (PCR) [2,3,13].

Acar et al. described dermoscopy as a non-invasive diagnostic tool in evaluating skin-pigmented lesions, inflammatory and non-inflammatory [18]. The dermoscopic features of sporotrichosis have not previously been reported. These features were numerous structure-less white areas in a lobular arrangement Sporothrix infection [18].

Treatment of sporotrichosis is mainly by itraconazole or amphotericin B [13]. Chen et al. produced therapeutic efficiency by inducing a humoral immune response for sporotrichosis treatment in an infected mouse model. This method can be attempted to substitute antifungal treatment when there is resistance to these drugs or in the presence of liver disease [19].

One case of a breast sporotrichosis infection, like our patient, was reported by a group from Brazil for a 73-year-old healthy lady. She presented with breast skin ulcers, nipple destruction, and pouring pus. The findings were suggestive of breast cancer. She had a history of cat scratches a few weeks earlier, and the fungus was isolated. She responded very well to itraconazole. The course was for seven months [3].

In 2015, Velazco et al. from Arizona also reported necrotizing cutaneous breast fungal infection in a patient with breast implants. The patient was 50 years old with a history of breast cancer and autologous tissue reconstruction. She presented with a skin necrotic infection one year after implant augmentation of the reconstruction. Pathological examination of debrided breast tissue revealed numerous fungal hyphae in the epidermis, dermis, and adipose tissue. She was started on voriconazole. Five months later, the patient presented with a skin lesion over her back, and Sporothrix species were isolated indicating disseminated disease [20].

## Conclusions

Sporotrichosis of the breast is rare in Bahrain, the Arabian Gulf, and other parts of the world. The patient was a middle-aged, healthy female who was not malnourished, immune-compromised, or of low socio-economic class. The only explanation for the source of the fungus in our patient is the fertilizer she used in her garden. This is the first case reported in Bahrain by reviewing the central lab registry in Bahrain. It was also not reported in Kuwait and other Arabian Gulf countries.

Whether our case worsened secondary to the COVID-19 infection or vaccine remains a controversial issue. The diagnosis of breast sporotrichosis needs a high index of clinical suspicion and includes a fungal culture of the biopsy and histopathology. Although itraconazole is the drug of choice for sporotrichosis, our patient response was slow. The aggressiveness of the disease requires aggressive treatment, like resection of the remaining inflamed area, to avoid the recurrence of the infection after withdrawal of the treatment.
